# Mechanical and Thermal Properties of Polypropylene Composites Reinforced with Lignocellulose Nanofibers Dried in Melted Ethylene-Butene Copolymer

**DOI:** 10.3390/ma7106919

**Published:** 2014-10-09

**Authors:** Shinichiro Iwamoto, Shigehiro Yamamoto, Seung-Hwan Lee, Hirokazu Ito, Takashi Endo

**Affiliations:** 1 Biomass Refinery Research Center, National Institute of Advanced Industrial Science and Technology (AIST), 3-11-32, Kagamiyama, Higashihiroshima, Hiroshima 7390046, Japan; E-Mails: yamamoto.shige@live.jp (S.Y.); t-endo@aist.go.jp (T.E.); 2 Department of Forest Biomaterials Engineering, College of Forest and Environmental Sciences, Kangwon National University, 1, Chuncheon 200701, Korea; E-Mail: lshyhk@hotmail.com; 3 TOCLAS Corporation, 1370, Nishiyamatyou, Nishiku, Hamamatsu, Shizuoka 4328001, Japan; E-Mail: Hirokazu_Ito@toclas.co.jp

**Keywords:** lignocellulose nanofiber, polypropylene, impact strength, ethylene-butene copolymer, wood flour, master batch

## Abstract

Lignocellulose nanofibers were prepared by the wet disk milling of wood flour. First, an ethylene-butene copolymer was pre-compounded with wood flour or lignocellulose nanofibers to prepare master batches. This process involved evaporating the water of the lignocellulose nanofiber suspension during compounding with ethylene-butene copolymer by heating at 105 °C. These master batches were compounded again with polypropylene to obtain the final composites. Since ethylene-butene copolymer is an elastomer, its addition increased the impact strength of polypropylene but decreased the stiffness. In contrast, the wood flour- and lignocellulose nanofiber-reinforced composites showed significantly higher flexural moduli and slightly higher flexural yield stresses than did the ethylene-butene/polypropylene blends. Further, the wood flour composites exhibited brittle fractures during tensile tests and had lower impact strengths than those of the ethylene-butene/polypropylene blends. On the other hand, the addition of the lignocellulose nanofibers did not decrease the impact strength of the ethylene-butene/polypropylene blends. Finally, the addition of wood flour and the lignocellulose nanofibers increased the crystallization temperature and crystallization rate of polypropylene. The increases were more remarkable in the case of the lignocellulose nanofibers than for wood flour.

## 1. Introduction

Wood is the most abundant biomass resource and has attracted considerable attention as a reinforcement filler for plastics. Owing to the high stiffness of wood, wood-based fillers can be used to increase the Young’s modulus and strength of general-purpose thermoplastics such as polypropylene (PP). Further, there have been significant advances in the development of wood-plastic composites [1,2].

Wood flour has been previously fibrillated into lignocellulose nanofibers (LCNFs) by mechanical disintegration using wet-state disk mill [3] and twin-screw extruder [4,5]. The obtained LCNFs exhibited a large specific surface area and were 20 nm in width in the most disintegrated area. LCNFs can be used as a nanoscale wood filler in PP composites. However, the dispersion of the hydrophilic LCNFs in PP composites is difficult, since LCNFs have a large specific surface area and readily form aggregations. It has been reported that the dispersion of freeze-dried LCNFs can be improved by solid-state shear pulverization [6]. The increase in the degree of dispersion improved the mechanical properties of the LCNF-reinforced PP composites.

Since LCNFs are prepared by wet-state fibrillation treatments, a drying process is necessary to produce LCNF composites. Although freeze drying is a useful method to reduce the formation of LCNF aggregations, it requires vacuum-like conditions and long processing times. There is also the issue of high cost with regard to using freeze drying on an industrial scale. The ideal drying method would involve evaporating the water in the LCNF suspension by heating. Thus, when mixing LCNFs and PP, the simplest drying process is likely to evaporating the water from the LCNF suspension in melted PP at a temperature higher than its melting point (150 °C). Since water is intensely evaporated in melted PP, LCNFs would form terrible aggregations.

The ethylene-butene copolymer (EBC), which is categorized as an elastomer, acts as an impact modifier for PP [7,8]. Low-butane-content EBC is immiscible with PP, but can be dispersed well in it. The impact strength of PP is enhanced by the addition of EBC, owing to its softness. However, the stiffness of PP decreases after the addition of EBC. The melting point of EBC is 70 °C and lower than that of PP. This suggests that the LCNF suspension can be dried during compounding with melted EBC at temperatures lower than the melting point of PP. Thus, the formation of LCNF aggregations can be reduced by slowly evaporating the water at temperatures close to its boiling point in comparison with the compounding of the LCNF suspension and melted PP.

The aim of this study was to develop PP composites reinforced by heat-dried LCNFs. The LCNF suspension was dried during pre-compounding with EBC at 105 °C to reduce the formation of LCNF aggregations. This mixture was used as a master batch for producing the final PP composites. That is to say, PP/EBC/LCNF composites were prepared by compounding PP and the master batch. In addition, maleic anhydride-grafted PP (MAPP) was added to improve the interfacial adhesion properties between the LCNFs and matrix polymer. The MAPP reacts with the hydroxyl groups of the wood filler and acts as a compatibilizer [9]. The composites were prepared by injection molding. Their thermal and mechanical properties were measured. Further, through a comparison of LCNFs and wood flour composites, the effects of the fibrillation of wood flour on the properties of composites were investigated.

## 2. Results and Discussion

### 2.1. Observation of Lignocellulose Nanofibers and Composites

**Figure 1:**
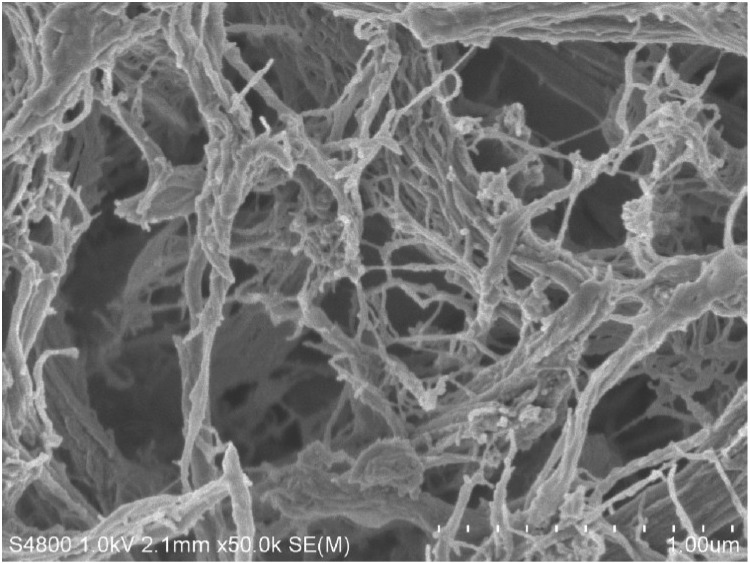
Scanning electron micrographs of the lignocellulose nanofibers.

Figure 1 shows a scanning electron microscopic (SEM) image of the LCNFs, which were prepared from wood flour by wet-disk milling. It can be seen that the wall structure of the wood cells had disintegrated into fibers less than 1 μm in thickness. The SEM image shows that the fibers were 20 nm in thickness in the area with the highest degree of disintegration. In previous studies, using nitrogen gas adsorption measurements, the specific surface area of LCNFs was found to be 106 m^2^/g [6], a value that is similar to the one reported for cellulose nanofibers [10]. The specific surface area of the fibers of untreated wood flour was 8 m^2^/g. That the specific surface area of the LCNFs was 13 times higher than that of the wood flour fibers. It indicated the effectiveness of wet-disk milling in fibrillating the wood flour.

**Figure 2:**
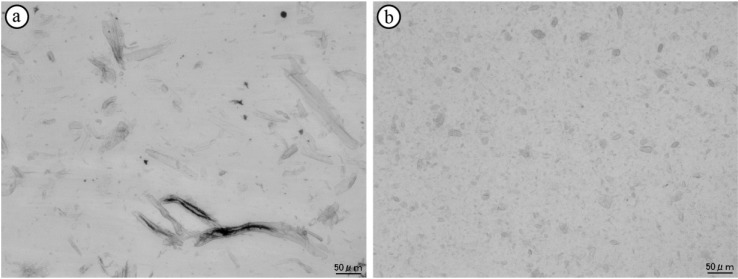
Optical micrographs of (**a**) wood flour and (**b**) lignocellulose nanofiber composites.The contents of wood flour and the lignocellulose nanofibers were both 5 wt%.

Figure 2 shows optical micrographs of the wood flour and LCNF composites. The original shape of the wood flour fibers can be observed in the composites (Figure 2a). The LCNFs in the composites were smaller in diameter (less than 20 μm) than the wood flour fibers (Figure 2b). Further, as can be seen from the images, the complete prevention of aggregation of the LCNFs could not be achieved by drying and the simultaneous compounding of the LCNF suspension in melted EBC at 105 °C. However, the degree of LCNF dispersion was improved, compared to the case in composites prepared using freeze-dried LCNFs in a previously reported study [6]. Because evaporating the water during compounding requires lesser time and consumes lesser energy than does freeze drying, the fabrication process employed in this study should be suitable for the industrial production of composites.

### 2.2. Mechanical Properties

**Figure 3:**
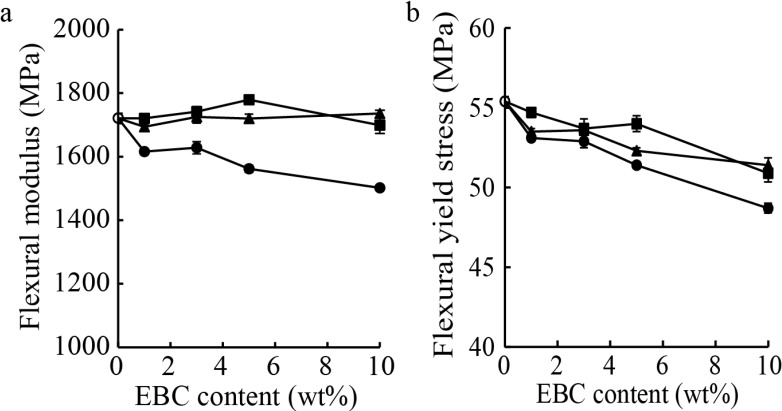
(**a**) Flexural moduli and (**b**) yield stresses of neat polypropylene (PP) (○), the PP/ethylene-butene copolymer (EBC) blends (●), and the wood flour (■) and lignocellulose nanofiber (LCNF) (▲) composites.

Figure 3 shows the flexural moduli and yield stresses of PP, the PP/EBC blends, and the prepared composites. Since EBC is softer than PP, the flexural moduli and yield stresses of the PP/EBC blends decreased with an increase in their EBC content. The wood flour and LCNF composites showed significantly higher flexural moduli (Figure 3a) and higher yield stresses (Figure 3b) than those of the PP/EBC blends for the same EBC contents. Furthermore, both composite types showed higher flexural moduli than that of neat PP. It was found that wood flour and the LCNFs acted as effective reinforcement fillers. Further, there was no significant difference in the flexural mechanical properties of the wood flour and LCNF composites.

**Figure 4:**
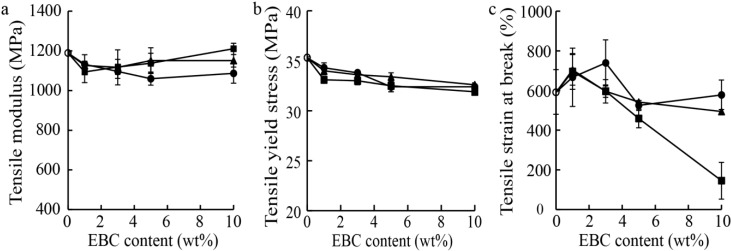
(**a**) Tensile moduli, (**b**) stresses and (**c**) strains at break of neat PP (○), the PP/EBC blends (●), and the LCNF (▲) and wood flour (■) composites. The composites contained 1 wt% maleic anhydride-grafted PP (MAPP).

Figure 4 shows the tensile moduli, yield stresses, and strains at break of PP, the PP/EBC blends, and the fabricated composites. The effects of the addition of EBC on the tensile modulus were not significant (Figure 4a). Further, the reinforcing effects of the addition of wood flour and the LCNFs on the tensile modulus and yield stress were also not significant. However, the strains at break of the composites containing 10 wt% wood flour were markedly lower than those of the other composites (Figure 4c).

The aspect ratio of the LCNFs was larger than that of the wood flour fibers, as shown in Figure 1 and Figure 2a. It has been reported that using fillers with a larger aspect ratio in composites results in a higher reinforcing efficiency, owing to the effective transfer of stress from the matrix to the filler [11,12,13]. However, the reinforcing effects of the LCNFs and wood flour on the moduli and stresses determined by the flexural and tensile tests were almost similar. This indicated that the LCNFs in the composites formed particle-shaped aggregations instead of remaining in the form of the original, large-aspect-ratio fibers.

**Figure 5:**
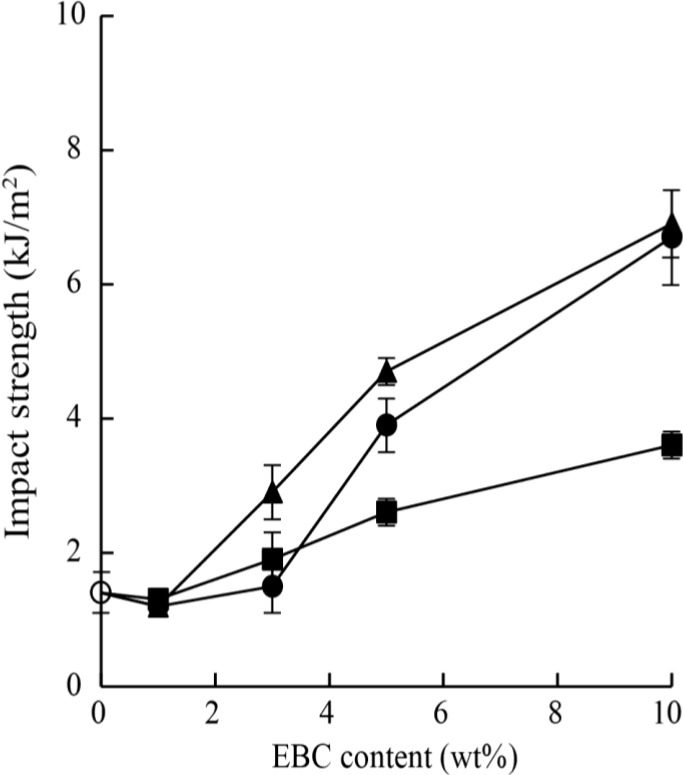
Izod impact strengths of neat PP (○), the PP/EBC blends (●) and the LCNF (▲) and wood flour (■) composites. The composites contained 1 wt% MAPP.

**Figure 6:**
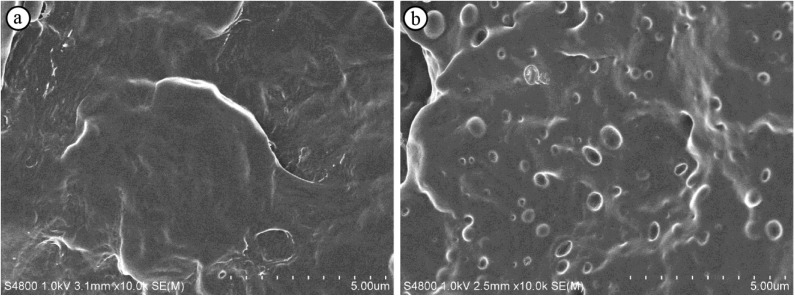
Scanning electron micrographs of the fractural surfaces after the impact tests of (**a**) the neat PP and (**b**) PP/EBC blend (90/10).

Figure 5 shows the Izod impact strengths of neat PP, the PP/EBC blends, and the fabricated composites. The addition of EBC in amounts greater than 5 wt% significantly increased the impact strength of PP. The LCNF composites showed the same or higher impact strengths than those of the specimens of the PP/EBC blends with the same EBC contents. However, the composites containing more than 5 wt% wood flour exhibited lower impact strengths than did the PP/EBC blends and LCNF composites with the same EBC content. Figure 6 shows SEM images of the fractural surfaces of neat PP and PP/EBC blends (90/10) after the impact tests. EBC is immiscible with PP, but disperses well in it, and formed domains smaller than 500 nm in the PP matrix. The soft EBC domains acted as impact stress absorbers, resulting in an increase in the impact strength.

**Figure 7:**
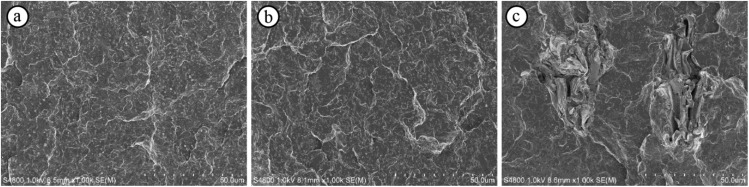
Scanning electron micrographs of fractural surfaces after the impact tests of the specimens of the (**a**) PP/EBC blend (90/10) and the (**b**) LCNF and (**c**) wood flour composites. The wood flour and LCNF contents of the composites were 10 wt%.

Figure 7 shows SEM images of the fractural surfaces after the impact tests of the PP/EBC blends and the wood flour and LCNF composites. Submicron sized-domains of EBC were observed in all the samples. Further, there was no significant difference between the surface morphologies of the specimens of the PP/EBC blends and those of the LCNF composites (Figure 7a,b). The LCNFs in the composites were probably embedded within the PP matrix. On the other hand, wood flour was found on the fractural surfaces of the specimens of the corresponding composites (Figure 7c). This indicated that the interface between the wood flour fibers and the PP matrix underwent delamination and that this is what resulted in the brittle fracturing of these specimens. That is to say, this is the reason these specimens exhibited low impact strengths.

The brittle fracture behavior of the specimens of the wood flour composites is shown along with the results of the tensile tests. When the composites were deformed by a large degree, voids formed owing to the delamination of the interface between the filler (wood flour and LCNFs) and the matrix (PP and EBC). The void size depended on the filler size, meaning that wood flour created larger voids in the composites than did the LCNFs. The larger voids resulted in fractures occurring more readily. This also explains why the specimens of the wood flour composites were more brittle than the specimens of the LCNF composites.

Thus, it can be surmised that LCNFs as a filler material are more effective in preventing the brittle fracture of composites than wood flour. The ductile fractural behavior of the specimens of the LCNF composites indicated that their impact strength was twice as high as that of the specimens of the wood flour composites with a filler content of 10 wt%. Further, the addition of the LCNFs not only countered the decrease in the flexural stiffness of PP resulting from the addition of EBC, but also increased its flexural stiffness.

### 2.3. Thermal Properties

The thermal properties of PP, the PP/EBC blends, and the wood flour and LCNF composites as measured by differential scanning calorimetry (DSC) are shown in Table 1. There were no significant differences in the melting temperatures (*T*_m_) and heats of fusion (∆*H*_m_) of PP, the PP/EBC blends, and the composites during the first heating stage. On the other hand, the crystallization temperature (*T*_c_) of PP decreased during the cooling stage after the addition of EBC. Furthermore, the addition of wood-based fillers increased the *T*_c_ of the PP/EBC blends with the same EBC contents. This result was attributable to the nucleation-inducing ability of the wood-based fillers. It has been reported that the addition of a wood-based filler increased not only the *T*_c_ of PP during the nonisothermal cooling stage, but also its isothermal crystallization rate [14,15]. The surfaces of the wood-based fillers initiated nucleation at a higher rate, resulting in the growth of a greater number of crystals. Thus, this nucleation-inducing ability of the wood-based fillers increased the *T*_c_ of the composites. Furthermore, since the interfaces between the LCNFs and PP in the composites were larger in area than those in case of the wood flour-based composites, the LCNFs acted as more effective nucleation agents than did wood flour. This resulted in the *T*_c_ of the LCNF composites being higher than those of the wood flour composites.

**Table 1:** Melting temperatures (*T*_m_), heats of fusion (∆*H*_m_), crystallization temperatures (*T*_c_), crystallization enthalpies (∆*H*_c_), and isothermal crystallization half-times at 135 °C (τ_1/2_) of PP, the PP/EBC blends, and the fabricated composites.

Constituents (wt%)					*T*_m_ (°C)	∆*H*_m_ (J/g)	*T*_c_ (°C)	∆*H*_c_ (J/g)	τ_1/2_ (min)
PP	EBC	Wood flour	LCNFs	MAPP					
100	0	0	0	0	160	90	121	95	11.8
95	5	0	0	0	161	89	119	94	20.2
90	10	0	0	0	160	88	119	95	19.5
89	5	5	0	1	161	87	121	98	7.9
79	10	10	0	1	158	88	122	97	7.1
89	5	0	5	1	159	87	124	98	4.3
79	10	0	10	1	159	89	125	98	3.3

In the case of the isothermal crystallization at 135 °C, the addition of EBC decreased the isothermal crystallization half-time (τ_1/2_) of PP, meaning that the isothermal crystallization of the PP/EBC blends was slower than that of PP. However, the addition of the wood-based fillers increased the crystallization rates of PP and the PP/EBC blends. In addition, the LCNF composites showed higher crystallization rates than did the wood flour composites. These increases in the isothermal crystallization rates can also be explained by the reason used to describe the results of the nonisothermal crystallization analysis. In a previous study [6], we had found that the improved dispersion of LCNFs in PP through solid-state shear pulverization induced fast isothermal crystallization, owing to the increase in the area of the filler-matrix interface. In this study, too, the increase in the interface area because of the fibrillation of wood flour caused the isothermal crystallization rate of the LCNF composites to be higher than that of the wood flour composites.

## 3. Experimental Section

### 3.1. Materials

Wood flour with a particle size of less than 0.2 mm (Japanese cypress; soft wood) was used. The EBC sample used (TAFMER DF810; melt flow rate (MFR) at 230 °C is 2.2 g/10 min) was provided by Mitsui Chemicals Co., Tokyo, Japan. PP (NOVATEC MA3; isotactic polypropylene homopolymer; MFR at 230 °C is 11 g/10 min; Japan Polypropylene Co., Tokyo, Japan) and MAPP (Kayabrid 005PP; graft ratio of maleic anhydrate is 4 wt%; Kayaku Akzo Co., Tokyo, Japan) were used as the matrix polymer and compatibilizer, respectively.

### 3.2. Lignocellulose Nanofibers

A water suspension of the wood flour (3 wt%) was passed through a disk mill (MKCA6-3, Masuko Sangyo Co., Saitama, Japan) equipped with two grinding stones that had a rotational speed of 1800 rpm. The gap between the grinding stones was narrowed to 150 μm from the initial contact distance. This fibrillation treatment was repeated 15 times. The obtained LCNF suspension was concentrated to 8 wt% using a centrifuge for 10 min at 14,000 g.

### 3.3. Composite Preparation

EBC (40 g) was compounded using a batch-type kneader (4C150 Laboplastomill with R60 screws, Toyo Seiki Seisaku–sho Co., Tokyo, Japan) at 105 °C and a rotating speed of 10 rpm. The LCNF suspension (solid content was 40 g) was added drop by drop into the melted EBC in the kneader for 3 h. After that, the kneading was continued for 20 min at 105 °C and a rotating speed of 40 rpm to evaporate the water completely. The obtained EBC and LCNF (1:1 weight ratio) mixture was used as the master batch for preparing the LCNF-reinforced PP composites. The master batch for the wood flour-reinforced composites was prepared by compounding dried wood flour and EBC using the kneader for 20 min at 105 °C and a rotating speed of 40 rpm.

The master batches were melt-compounded with PP and MAPP using a twin-screw extruder (4C150 Laboplastomill with 2D15W screws, Toyo Seiki Seisaku–sho Co., Tokyo, Japan) at 170 °C and 30 rpm, and were cut into pellets. The pellets were then molded using an injection molder (Babyplast 6/10P, Cronoplasto, S. L., Barcelona, Spain); the temperatures for extrusion and injection were 180 and 190 °C, respectively. The injection pressure was 13 MPa, and the mold temperature was equal to the room temperature (approximately 25 °C). The composites were injected into dumbbell-like and bar-shaped molds. The dimensions of the dumbbell-like and bar-shaped specimens were 4 × 2 × 20 mm^3^ (central rectangular part) and 10 × 4 × 58 mm^3^, respectively. The amount of the respective master batch in the final composites was 2 to 20 wt%, indicating that the amount of EBC (1 to 10 wt%) in the final composites was same as that of the wood-based fillers. The amount of MAPP in the composites was 1 wt%. For comparison, samples of neat PP and PP/EBC blends (amount of EBC was 1 to 10 wt%) were also prepared by extrusion and injection molding under similar conditions.

### 3.4. Scanning Electron Microscopy

The morphology of the LCNFs and the fracture surfaces of the injection-molded samples were observed using SEM (S-4800, Hitachi High-Technologies Co., Tokyo, Japan). The LCNF suspension was solvent-exchanged with t-butyl alcohol and then freeze dried, so that the morphology of the fibers was maintained. The fracture surfaces were obtained through impact tests. The dried LCNFs and fracture surfaces were coated with osmium by vapor deposition (Neoc-ST, MEIWAFOSIS Co., Tokyo, Japan) before the observations.

### 3.5. Mechanical Properties

Tensile and flexural tests were performed to determine the mechanical properties of the composites using a mechanical tester (AGS-5kNG, Autograph, Shimadzu Co, Kyoto, Japan). Three-point flexural tests were performed on the bar-shaped injection-molded samples at a crosshead speed of 5 mm/min; the span length for the tests was 50 mm. The dumbbell-like injection-molded samples were subjected to tensile tests. The tensile tests were performed at a crosshead speed of 10 mm/min.

The notched Izod impact strengths of the bar-shaped injection-molded samples were measured using a universal impact tester (No. 258-D, Yasuda Seiki Seisakusho Co., Hyogo, Japan). The fabricated notches were 0.25 mm in radius and 2 mm in depth.

### 3.6. Thermal Properties

Differential scanning calorimetry (DSC) measurements were performed using a Pyris 1 DSC calorimeter (Perkin–Elmer Co., Waltham, MA, USA). The samples were first heated from 30 to 200 °C at a rate of 10 °C/min and held at 200 °C for 1 min. They were then cooled to 50 °C at a rate of 10 °C/min and held at 50 °C for 1 min. Next, they were again heated to 200 °C at a rate of 10 °C/min and held at 200 °C for 1 min. Finally, they were quenched to 135 °C at a rate of 50 °C/min to determine their τ_1/2_ values. The temperatures corresponding to the exothermic and endothermic peaks in the first heating step and the cooling step were called *T*_m_ and *T*_c_, respectively.

∆*H*_m_ and ∆*H*_c_ values were determined from the areas of the melting and crystallization peaks, respectively. The ∆*H*_m_ and ∆*H*_c_ values were converted on the basis of the PP weight ratio of the composites.

## 4. Conclusions

In this study, we investigated the mechanical and thermal properties of PP composites reinforced with heat-dried LCNFs. The copolymer EBC was pre-compounded with a LCNF suspension to obtain a master batch. At the same time, the water in the LCNF suspension was evaporated by heating. The final composites were produced by the compounding of the master batch and PP. The formation of aggregations of the LCNFs could not be prevented completely. However, it was reduced by the two-step process adopted in this study.

The addition of the LCNFs not only enhanced the flexural stiffness of PP/EBC blends but also increased the impact strength of the composites such that it was twice as high as that of the wood flour composites for a filler content of 10 wt%. The addition of wood flour and the LCNFs both increased the crystallization temperature and crystallization rate of PP. However, the increase was greater in the case of the LCNFs than in the case of wood flour. On the other hand, the addition of wood flour and the LCNFs had no significant effect on the melting behavior of PP.
